# GuideNav: User-Informed Development of a Vision-Only Robotic Navigation Assistant for Blind Travelers

**DOI:** 10.1145/3757279.3785625

**Published:** 2026-03-16

**Authors:** Hochul Hwang, Nicholas A. Giudice, Soowan Yang, Sunghoon Ivan Lee, Donghyun Kim, Jahir Sadik Monon, Joydeep Biswas

**Affiliations:** University of Massachusetts at Amherst, Amherst USA; University of Maine, Orono USA; DGIST, Daegu Republic of Korea; University of Massachusetts at Amherst, Amherst USA; University of Massachusetts at Amherst, Amherst USA; University of Massachusetts at Amherst, Amherst USA; The University of Texas at Austin, Austin USA

**Keywords:** Assistive Robotics, Guide Dog Robot, Navigation

## Abstract

While commendable progress has been made in user-centric research on mobile assistive systems for blind and low-vision (BLV) individuals, references that directly inform robot navigation design remain rare. To bridge this gap, we conducted a comprehensive human study involving interviews with 26 guide dog handlers, four white cane users, nine guide dog trainers, and one O&M trainer, along with 15+ hours of observing guide dog–assisted walking. After de-identification, we open-sourced the dataset to promote human-centered development and informed decision-making for assistive systems for BLV people. Building on insights from this formative study, we developed GuideNav, a vision-only, teach-and-repeat navigation system. Inspired by how guide dogs are trained and assist their handlers, GuideNav autonomously repeats a path demonstrated by a sighted person using a robot. Specifically, the system constructs a topological representation of the taught route, integrates visual place recognition with temporal filtering, and employs a relative pose estimator to compute navigation actions—all without relying on costly, heavy, power-hungry sensors such as LiDAR. In field tests, GuideNav consistently achieved kilometer-scale route following across five outdoor environments, maintaining reliability despite noticeable scene variations between teach and repeat runs. A user study with 3 guide dog handlers and 1 guide dog trainer further confirmed the system’s feasibility, marking, to our knowledge, the first demonstration of a quadruped mobile system guiding a route in a manner comparable to guide dogs.

## 1 Introduction

The first step in developing mobile navigation assistants for blind and low-vision (BLV) travelers is identifying their most meaningful real-world needs. While commendable strides have been made in user-centered development of navigation systems [6, 26, 31, 33, 39, 40, 56, 63, 68], defining a navigation system that is truly practical for BLV individuals remains challenging. Building a navigation assistant for BLV travelers is not simply a matter of replicating autonomous driving systems—like a delivery robot using dense map-based navigation moving from point A to B on a prebuilt map [41]. Prior studies on navigation assistance showed that the level of assistance can vary widely, from mobility-only support (i.e., obstacle avoidance) to full autonomy (i.e., governing navigation direction) [56]. Other works [40, 68] investigated exploratory navigation setups that allow BLV travelers to visit new places (e.g., exhibitions) with additional scene description assistance. Studies on interactive navigation of handlers and guide dogs have also highlighted the importance of trust and a sense of agency as prerequisites for introducing autonomous navigation to BLV people [31].

Such prior research provides valuable insights into BLV navigation relating to robotic guides; however, many detailed technical questions remain unanswered: What is the proper sensor suite—can LiDAR be an appropriate option? What conditions (e.g., terrain types, weather, lighting) must the robot handle? How much distance should the system support for daily navigation? And is it valid to assume that users have prior knowledge of the places they navigate? Answering such questions requires multi-dimensional considerations. For example, evaluating the suitability of LiDAR depends on power consumption, onboard computing capacity, operating conditions, and cost. These factors, in turn, are shaped by BLV users’ daily navigation patterns: how far they typically walk, whether they commute in rain, fog, or snow, and the acceptable robot weight (which constrains battery and computer size).

These questions may never be fully resolved until a system is deployed to end users. Despite extensive research on spatial cognition and navigation in psychology, cognitive science [20], and HRI, resources capturing the lived practices of BLV travelers remain scarce. This limits the translation of theoretical insights into practical, human-centered navigation systems. By examining BLV users’ daily activities, commuting habits, common destinations, and limitations of current mobility aids, developers can reduce design errors. However, such open-source data remains minimal—a key obstacle for human-centered navigation research.

To bridge this gap, we conducted a formative study with interviews of 26 guide dog handlers, four cane users, nine guide dog trainers, and one O&M trainer, along with 15+ hours of observing how guide dog handlers navigate with their dogs. We examined how BLV individuals interact with guide dogs, which informed the design of our pragmatic navigation assistant. The resulting dataset, GuideData, was carefully anonymized, organized for ease of use, and open-sourced to promote human-centered research and inform assistive system design for BLV people.

Building on insights from our formative study, we aimed to define an ideal navigation system to support the independent mobility of BLV individuals. We selected a quadruped robot platform for robust maneuverability across diverse terrains, and chose not to rely on GPS or LiDAR. LiDAR is costly in cost, compute, and weight, reducing operating time, and it is less reliable in adverse weather (e.g., rain, fog, snow) and around transparent (e.g., glass doors) or reflective surfaces (e.g., water puddles) common in daily environments. It also typically requires dense, prebuilt 3D maps, which are computationally and memory-intensive for embedded systems [54]. GPS, meanwhile, is unreliable indoors and in urban environments due to signal degradation and top-down measurement limitations.

**Figure 1:**
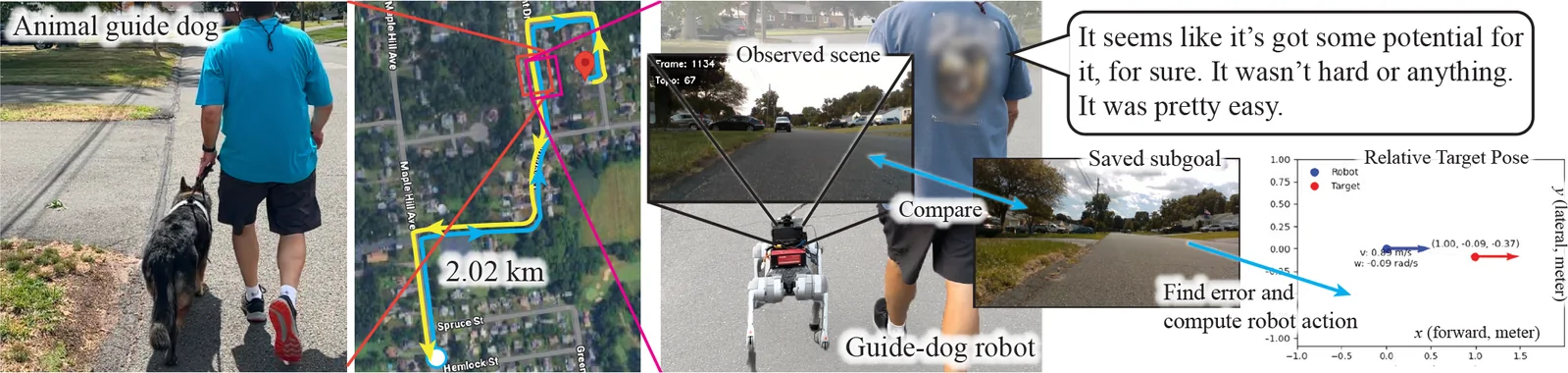
We developed a vision-only route following system considering technical requirements for practical guide robots. Our guide dog robot achieved kilometer-scale guiding with blind and low-vision (BLV) users. Robust path following performance and positive feedback from BLV users mark an important milestone toward the real-world deployment of guide dog robots.

Instead of metric map-based navigation [5, 18, 38], we propose GuideNav, following a Visual Teach-and-Repeat (VT&R) framework [23]. An operator first demonstrates a route by teleoperating the robot, after which the robot autonomously repeats the path for a BLV user. This framework aligns closely with how guide dogs are trained and work with their handlers when familiarized with the route. Since daily mobility often follows routine paths, we target these scenarios as a practical starting point. During the teach phase, the robot records images and builds a topological map by storing keyframes as image-based subgoals, enabling vision-only navigation with a single RGB camera (see Fig. 1).

While promising, VT&R is vulnerable to scene variation: changes in lighting or object layout can reduce keyframe retrieval accuracy and cause the robot to lose its path [23]. To improve robustness, we follow prior work [7, 66] by using visual place recognition (VPR) embeddings [1, 48] instead of raw pixel matching [13]. We further integrate Reloc3r [15] to estimate the robot’s relative pose to target keyframes and generate control commands (linear and angular velocities). Reloc3r outperforms dense feature-matching methods [19, 43, 65], which often suffer from degraded accuracy when depth information is unreliable (common in outdoor environments) or fail under scene variations.

Field tests show that our system achieves kilometer-scale, long-range autonomous navigation, surpassing prior guide robot demonstrations that relied on more complex sensing and mapping [6, 68]. GuideNav uses no metric maps, GPS, LiDAR, or depth sensing, keeping the platform lightweight and low-cost. By chaining short, visually guided segments, GuideNav avoids drift accumulation and adapts to environmental changes, remaining robust with modest computing resources and supporting long operating times.

In user studies, GuideNav successfully guided BLV participants along outdoor routes up to 2.02 km round trip. To our knowledge, this is the first kilometer-scale outdoor autonomous guiding demonstration for BLV users using a camera-only system. Participants found it easy to use, and trainers noted its reliability and distinct behavior from guide dogs. Workload and usability scores indicated low cognitive demand and high satisfaction, while highlighting areas for refinement and potential applications.

In summary, our contributions are threefold:

1. A large open-source dataset containing rich information about handler-guide dog interactions, real-world navigation challenges, and BLV travelers’ daily activities in navigational contexts.
2. A lightweight, visual teach-and-repeat framework that eliminates reliance on GPS, LiDAR, and depth sensing.
3. The first real-world validation of kilometer-scale, vision-only autonomous navigation with BLV participants on daily routes, measuring turning success rates, interventions, and collisions under diverse outdoor conditions.

## 2 Related Works

### 2.1 Existing Technological Solutions

*Un-actuated systems* include smart canes [9, 21, 60, 63, 69, 70, 74, 75, 84] and wearable or handheld devices [2, 12, 30, 34, 45, 55, 56, 59, 64, 85]. While lightweight and compact, they typically provide only limited navigation assistance—for example, notifying users of nearby obstacles or local paths, but no goal-directed navigation. More critically, the use of acoustic or vibrotactile feedback (rather than physical pulling or pushing) can increase distraction and mental fatigue, raising safety concerns because BLV individuals rely heavily on environmental sounds for situational awareness [4].

In contrast, *actuated systems* offer guidance more similar to sighted guides or guide dogs, enabling smoother collision avoidance, faster walking, and shorter travel distances [6, 10, 51]. Since the mid-1970s, most actuated systems have used wheeled robots [24, 26, 35, 37, 40, 42, 49, 50, 52, 67, 68, 72, 73], but wheels struggle with uneven terrain such as stairs and curbs [68], limiting real-world outdoor deployment.

Recent advances in quadruped robots have opened new opportunities for mobility assistance. Quadruped robots provide robust mobility across uneven and heterogeneous terrain [8, 27, 53, 81], but many studies underemphasize user needs due to limited understanding of how guide dogs and their handlers work. For instance, [8, 81] explored the use of a soft leash to pull a person, whereas both our formative study and prior literature [26, 31, 32] emphasize that handlers require a rigid harness handle to perceive immediate feedback on the guide dog’s motion for safety. Similarly, some studies positioned the handler behind the robot during navigation [6, 27], whereas in practice guide-dog handlers walk beside and one step behind the dog’s front feet, enabling them to assess the environment when the dog stops for safety [31].

Overall, quadrupeds offer strong potential for assistive mobility, yet existing guide dog robot research lacks a comprehensive user-centered perspective. We aim to develop a pragmatic guide robot that matches—and ultimately surpasses—the capabilities of animal guide dogs, providing BLV individuals with a reliable option for independent navigation.

### 2.2 Vision-based Navigation and VT&R

While prior VT&R studies demonstrated kilometer-scale path following [23], they often relied on precise metric localization or expensive sensors. For example, many VT&R variants used LiDAR to register the robot’s pose [23]. More recently, there has been a major departure from such approaches, with new methods aiming to be lightweight and vision-centric. Fischer et al. [13] proposed a bio-inspired scheme that leverages odometry and minimal visual correction (sparse cues) to navigate reliably with low computational cost. However, methods employing naive template matching (e.g., Normalized Cross Correlation [44]) are fragile to visually similar patterns and often produce false matches under appearance ambiguities.

FFI-VTR [76] advanced this line of work by constructing a topological keyframe graph of feature descriptors, minimizing pixel-level feature distance between matches without requiring global localization. PlaceNav [66] further reframed VT&R subgoal selection as an image-retrieval task: it uses visual place recognition embeddings (CosPlace [1]) to pick the next topological subgoal and applies Bayesian filtering for temporal consistency [82]. While PlaceNav shares some similarity with our GuideNav in subgoal selection, GuideNav also employs a trained network for pose estimation, which enhances robustness to scene and lighting variations and enables reliable long-range navigation.

## 3 Formative Study Dataset

Existing qualitative research spans guide dog ownership [57, 79], interaction [11, 29, 86], navigation and communication [25, 47, 63, 80]. In parallel, developers of assistive mobility systems report participatory design [6, 39], experiments [14, 36, 46, 77], and observations [17, 32, 35]. However, few studies release rich demonstration videos [46] or HRI data [6, 36], and large-scale open qualitative resources—especially handler-guide dog observations and trainer/handler interviews—remain scarce [8, 27, 81].

To address this gap and inform the human-centered design of GuideNav, we conducted a formative study documenting interviews, navigation, video observations, the matching process, and blindfolded walking sessions. We open-sourced GuideData, a curated and de-identified dataset of 39 interview transcripts, 31 images, and 126 videos (15+ hours), available at https://guidedogrobot-navigation.github.io/.

### 3.1 Procedure

GuideData includes five data types with detailed documentation:

- **Interviews with guide dog handlers:** Semi-structured Q&A on daily interaction with guide dog (transcripts).
- **Interviews with guide dog trainers:** Semi-structured Q&A on guide dog training and deployment (transcripts).
- **Observation sessions of guide dog–assisted navigation:** Videos of handler-guide dog navigation on routine routes (videos).
- **Observation sessions of matching training:** Videos documenting how an experienced handler and a new guide dog undergo the initial familiarization process (videos).
- **Blindfolded walking sessions by the authors:** Under a guide dog trainer’s supervision, we conducted blindfolded walks with trained guide dogs to experience handler–guide dog interaction and key navigation cues needed for safe travel. The video recordings document dog-provided support (e.g., locating sidewalks) versus decisions requiring human judgment (e.g., assessing street-crossing safety), yielding rich evidence on how animal guide dogs deliver navigation assistance (videos).

### 3.2 Participants

GuideData includes semi-structured interviews with 11 male and 21 female BLV individuals, and observation sessions with seven guide dog users and one cane user. Participants had an average age of 64.63 years and roughly 24.21 years of experience using navigation aids, with the majority having experience with multiple guide dogs. We also include interviews and observation sessions with nine professional guide-dog trainers and one O&M specialist. Demographics are provided in Supplementary Material 1.

### 3.3 Data Postprocessing

To protect privacy, all video recordings are post-processed to blur participants’ faces. For observation videos and images, we applied an automatic face anonymization algorithm [16, 83] to obscure participants and visible license plates. All frames were carefully reviewed to correct any failures of the automated process using a video editing tool. Interview transcripts were de-identified by removing personally identifiable information. The dataset is released on Kaggle under a CC0 license. To our knowledge, GuideData is the first dataset combining interviews and real-world observations that captures how guide dogs assist navigation in practice, providing a foundation for designing robotic guide systems.

## 4 Technical Requirements for Guide Robots

Designing a practical guide robot for blind and low-vision travelers is uniquely challenging. Low employment rates demand cost-sensitive solutions, yet the robot must reliably operate across diverse real-world environments encountered in daily travel. Grounded in GuideData, this section translates formative insights from handlers and trainers into technical requirements for a vision-only guide robot. Hardware constraints (Section 4.1) emphasize practical deployment—long runtime, independence from unreliable GPS, and minimal sensor complexity—while navigation requirements (Section 4.2) follow Orientation & Mobility (O&M) principles such as straight-line travel and curb recognition. Our current GuideNav system reflects these needs through compact topological mapping and kilometer-scale route following, while obstacle avoidance remains an open challenge. We defer other aspects such as communication, personalization, and user interfaces [31] to future work.

### 4.1 Hardware Constraints

#### 4.1.1 Runtime and responsiveness.

The system must operate for at least two hours [31] at normal walking speeds (up to at least 1.4 m/s) [3], with all perception and control onboard to avoid network dependence. We propose that end-to-end (perception to control) latency must remain under 100 ms so that safety-critical commands (e.g., “stop,”) are executed promptly and trusted by the handler. Memory must support storage of large-scale maps.

#### 4.1.2 Form factor and weight.

The robot must pass through narrow doors, fit under seats, and be light enough for one person to lift when necessary [78], yet heavy and stable enough to safely halt an adult in emergencies. Legged or wheel–legged locomotion is preferred to negotiate curbs, stairs, and uneven terrain that are encountered in daily travels.

#### 4.1.3 Minimal Sensor Suite: Vision-Only Approach.

A forward-facing RGB camera is essential for capturing rich visual cues. In contrast, LiDAR – though common in guide robot research – adds cost, power draw, and vulnerability to weather (e.g., drizzle, fog, snow) that corrupt point-cloud returns which can be problematic as handlers may get caught in rain and snow (GH03, GH12). Handlers reported accidents caused by transparent hazards, breaking one’s own leg after falling from ice, or glass doors, which LiDAR often fails to detect. Similarly, GPS proved unreliable in dense urban areas where several participants reported not using GPS-based apps due to frequent signal loss. These findings underscore the rationale for a vision-only approach, as the most practical and scalable option for daily navigation.

### 4.2 Navigation Requirements for Guide Robots

Navigation for BLV travelers imposes requirements distinct from conventional mobile robotics. A guide robot must compactly encode long routes across varied terrains, adhere to orientation principles emphasized in mobility training, and reliably negotiate the diverse obstacles encountered in sidewalk environments. These requirements motivate lightweight, robust approaches such as Visual Teach and Repeat (VT&R) [23], where routes are compactly stored and repeated under changing conditions.

#### 4.2.1 Mapping and Perception.

Guide robots must represent long range (kilometer-scale) routes spanning both structured indoor corridors and unstructured outdoor paths (e.g., cracked sidewalks, curbs, stairs). Compact map representations (e.g., visual descriptors) are preferable to dense 3D point clouds for efficiency. Perception must remain reliable across lighting, weather, and seasonal changes, including transitions from daylight to low-light operation.

#### 4.2.2 Domain Knowledge for BLV Travel.

Navigation must integrate orientation and mobility (O&M) practices. For instance, before street crossings the robot should stop at curb edges within one stride, allowing the traveler to probe with their foot and decide when to proceed. O&M training emphasizes maintaining straight-line travel and executing turns only at right angles on command. In areas without sidewalks, teams often “shoreline” the left road edge to preserve orientation – patterns the robot should replicate.

#### 4.2.3 Obstacle Avoidance.

Trainers specify that safe clearance requires at least 0.9 m laterally and 1.8 m vertically. Beyond standard obstacles, guide robots must detect overhanging (branches, signs, wires) and transparent hazards (glass panes, puddles, ice) that often challenge vision systems. Handler interviews also highlighted everyday obstructions – manholes, construction equipment, and parked cars – that block pedestrian paths and must be negotiated.

## 5 Proposed GuideNav Architecture

**Figure 2:**
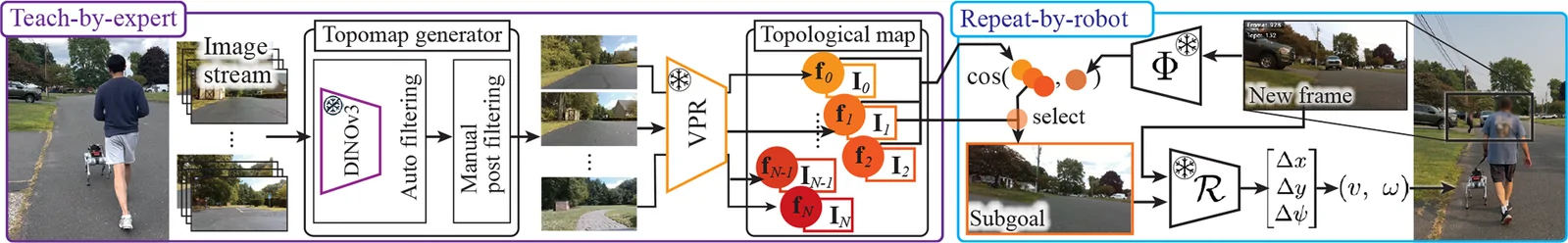
GuideNav teach-and-repeat framework. In the *teach* phase, an expert teleoperates the robot along a route while the system records a sequence of images. The topomap generator then builds a topological map by selecting keyframes. In the *repeat* phase, GuideNav performs visual place recognition by matching current observation features $f_{t}$ (extracted by Φ) to the topomap via cosine similarity to select a subgoal, then estimates relative pose using the pose estimator ℛ. GuideNav achieved kilometer-scale navigation under varying scene conditions and was evaluated by stakeholders, who provided positive feedback on its potential to support mobility assistance for BLV individuals.

Unlike monolithic end-to-end policies, our approach integrates modular components—topological mapping, visual place recognition, relative pose regression, and control—to achieve kilometer-scale outdoor route following on embedded hardware (see Fig. 2).

### 5.1 Problem Formulation

We define the guide dog route following problem as enabling a robot to safely repeat expert-taught pedestrian routes using only onboard sensing and compute. Formally, the robot state lies in $SE(2),x_{t}=\left(p_{t},\psi_{t}\right)$, where $p_{t}\in R^{2}$ is position and $\psi_{t}$ is orientation. At each timestep, the robot observes $I_{t}$ through a camera.

During the **teach phase**, an expert demonstrates a safe route $\Gamma ={\left\{I_{k}\right\}}_{k=1}^{M}$, consisting of M temporally dense observations. From the demonstration, we construct a sparse topological map $ℳ={\left\{\left(I_{i},f_{i}\right)\right\}}_{i=1}^{N}$, where N≪M. Each keyframe $I_{i}\in R^{H\times W\times 3}$ is selected from the demonstration sequence Γ, and $f_{i}$ denotes its visual embedding extracted by a place recognition encoder. Keyframe selection is performed using our topological map generator with optional manual filtering, as detailed in Section 5.2.

During the **repeat phase**, the estimates the most likely subgoal $I_{g_{i}}\in ℳ$ and estimates the relative transformation $\xi_{t\to g_{i}}\in se(2)$. This relative pose is converted into velocity commands $\left(v_{t},w_{t}\right)$ under a nonlinear feedback controller, producing safe closed-loop motion along the expert-taught route.

GuideNav functions more like a global planner, autonomously following expert-taught subgoals without GPS or dense metric maps. By design, the system does not include a dedicated local planner, but its modular architecture allows one to be seamlessly integrated for detours or dynamic avoidance when required. This balance of simplicity and modularity ensures both efficiency on embedded hardware and adaptability for future extensions.

### 5.2 Topological map generator

Each collected frame $I_{t}$ is encoded using the DINOv3 foundation model [62] ℰ into an $\ell_{2}$-normalized global descriptor $z_{t}=ℰ\left(I_{t}\right)$. We select keyframes with an *adaptive selector* that jointly enforces (1) minimum temporal spacing, (2) appearance diversity over a recent buffer via cosine similarity, and (3) adaptive similarity thresholds that adjust based on trajectory progress. This embedding-driven approach yields compact yet discriminative topological maps, retaining only a small fraction of frames while improving robustness under viewpoint/illumination changes and reducing memory footprint (e.g., ~24 MB for 1 km travel). A human operator may filter low-quality keyframes (e.g., motion-blurred), though this step could be automated in future work.

### 5.3 Visual place recognition with temporal consistency

Each incoming image $I_{t}$ is encoded by CosPlace [1] into a normalized descriptor $f_{t}=\Phi \left(I_{t};\theta \right)\in R^{512}$. Cosine similarity enables efficient retrieval of candidate subgoals. To prevent erratic frame switching, we enforce temporal consistency through a belief update over topological states, following the approach of Suomela et al. [66]. Based on observation, this yields smoother subgoal selection and mitigates the kidnapping problem [71].

### 5.4 Direct relative pose estimation

Given a current frame $I_{t}$ and retrieved subgoal $I_{g_{i}}$, we estimate their relative pose using ReLoc3r [15] as our pose regression network ℛ:

(1) $$ℛ\left(I_{t},I_{g_{i}};\Theta \right)=T_{t\to g_{i}}\in SE(3),$$

with $T_{t\to g_{i}}=\left[\begin{array}{rr} R & t\\ 0^{⊤} & 1 \end{array}\right]$. The transform is projected onto the ground plane to yield

(2) $$\xi =[\Delta x,\Delta y,\Delta \psi {]}^{⊤}\in R^{3}$$

a compact 2D displacement and yaw representation directly consumed by the controller. Rather than relying on Perspective-n-Point (PnP) [22], which requires dense feature matching and is vulnerable to low texture and depth errors, using a direct regression approach provides robust pose estimates suitable for long-range navigation.

### 5.5 Goal Position to Velocity Commands

We model the robot with unicycle kinematics, where the relative pose $\xi ={\left[\Delta x,\Delta y,\Delta \psi \right]}^{⊤}$ is expressed in polar form:

(3) $$\rho =\sqrt{\Delta x^{2}+\Delta y^{2}},\;\alpha =arctan\;2(\Delta y,\Delta x),\;\beta =\psi -\alpha ,$$

with ρ the goal distance, α the heading error, and β the orientation misalignment. A nonlinear feedback law regulates these terms:

(4) $$v_{raw}=v_{max}\left(1-e^{-k_{\rho }\rho }\right),$$

(5) $$w_{raw}=k_{\alpha }\alpha +k_{\beta }\beta ,$$

where $\left(k_{\rho },k_{\alpha },k_{\beta }\right)$ are control gains. For stability, we further apply three heuristics: reduced speed near the goal, velocity damping under large heading errors, and amplified orientation correction in final alignment. Finally, coordinated scaling enforces velocity limits, yielding smooth, bounded trajectories consistent with unicycle dynamics and reliable teach-and-repeat navigation.

## 6 Evaluation of GuideNav Components

**Figure 3:**
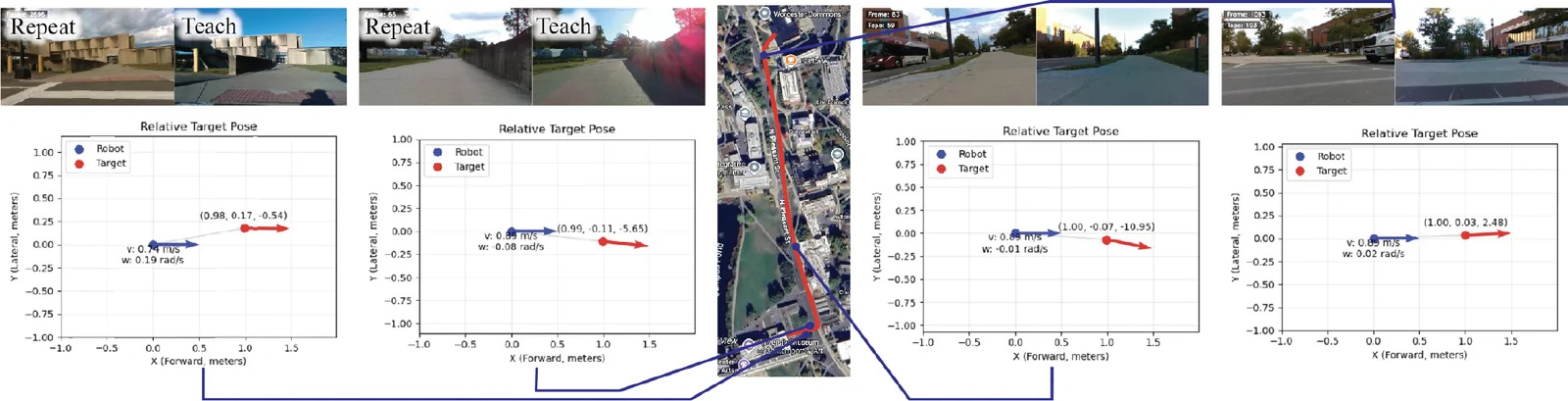
GuideNav outputs during guidance of H02, showing topological map node prediction and relative pose estimation. The system correctly predicts the node despite drastic lighting changes and unseen objects, and maintains accurate pose estimation.

We evaluated GuideNav in both indoor and outdoor environments to assess the robustness of its key components. As shown in Fig. 3, the topological map maintained reliable node prediction under lighting changes and partial occlusions.

### 6.1 Topological Map Node Prediction

We first examined the effect of different keyframe selection methods on route following. Specifically, we compared a simple distance-based heuristic with topological maps constructed from visual embeddings. Learning-based embeddings consistently improved repeat reliability over the heuristic, with DINOv3 providing the most robust localization across varied lighting and viewpoint conditions.

### 6.2 Relative Pose Estimation

We next compared direct relative camera pose regression with a feature-matching + PnP pipeline. While dense matching can yield accurate correspondences, it frequently failed under textureless surfaces and extremely bright conditions. In contrast, direct regression achieved higher stability, lower failure rates, and smoother control signals, making it better suited for long-horizon outdoor navigation.

## 7 Full System Evaluation with Stakeholders

We evaluated our full system in realistic contexts through two complementary studies. First, we conducted a field study with three BLV individuals, all experienced guide dog handlers. Second, we replicated an official guide dog evaluation by testing the robot in a standard training environment: a blindfolded guide dog trainer served as the user, while another trainer acted as evaluator. Our objectives were twofold: (1) to assess the effectiveness and acceptance of the guide dog robot among BLV users, and (2) to benchmark performance against the criteria applied to animal guide dogs for real-world deployment.

### 7.1 Participants

We recruited three experienced guide dog handlers, each capable of independently navigating their local communities without a sighted guide. All relied primarily on a guide dog as their mobility aid. Eligibility criteria were (1) visual acuity at or below the legal blindness threshold defined in the Social Security Act §1614 [61], and (2) at least six months of experience working with a guide dog. Demographic details are shown in Table 1. H01 and H03 were totally blind (with H03 retaining light perception), while H02 had residual vision (20/2200 in one eye, lower in the other). In addition, three professional guide dog trainers were recruited, each with a minimum of four years’ experience. T01 observed the robot guiding a handler and provided evaluation as an expert bystander. T02 and T03 jointly completed the standard guide dog evaluation course, with T02 blindfolded in the role of user and T03 serving as evaluator.

### 7.2 Guide Dog Robot

Our guide dog robot is a compact, stand-alone quadrupedal platform designed for both indoor and outdoor route following. Our system is built on the Unitree Go2 robot equipped with a rigid harness handle that allows a rigid connection with the user. A single Intel RealSense D435i camera is used for GuideNav. Relative pose estimation is performed using Reloc3r-512 [15] with mixed-precision inference to achieve a high accuracy and inference speed. GuideNav runs at 5 Hz on a NVIDIA Jetson AGX Orin.

### 7.3 Procedure

1. **Guide dog demonstrations:** We observed handlers (H01-H03) walking familiar community routes with their guide dogs. H01 walked from the parking lot drop-off to her workplace (Lot ↔ Library). H02 began walking from home to the bus stop (Home → BusStop) but switched to another route (Dining ↔ FineArts) to avoid drawing neighbors’ attention. H03 walked a residential loop from home to the end of the sidewalk near a church which was over a kilometer one-way (Home ↔ Church). Additionally, trainer T02 walked a standard evaluation route blindfolded under evaluator T03’s supervision.
2. **GuideNav Teach (Data collection with robot):** Based on these observations, a researcher teleoperated the robot along each route, with minor adjustments for efficiency and safety (e.g., walking on the edge of a road instead of the center, by-passing unnecessary stops such as poles). The onboard camera recorded video to construct the topological map.
3. **GuideNav Repeat (Autonomous guiding trials):** GuideNav autonomously guided the users. Before each trial, handlers adjusted the harness handle (length and angle) and briefly practiced walking with the robot. The robot navigated to the destination on a single command. Users were notified just before the start and after the robot stopped when it reached the goal. One researcher logged data using a laptop for debug purposes (does not affect GuideNav performance), and another recorded video.
4. **Post-Trial Interviews and Surveys:** After each walk and a brief break, participants completed a semi-structured interview and workload/usability surveys. Interviews were audio-recorded, transcribed using a speech-to-text service [58]. Generative AI tools were used only to improve writing clarity.

### 7.4 Findings

All participants successfully reached the goal in their familiar daily routes within their local community with the guidance of the guide dog robot. The distance and the complexity of the daily routes varied as detailed in Table 2. For example, H03 successfully walked with the robot for over 2 km (1 km one-way) where he usually goes for a walk with his guide dog. H02 in the other hand, walked for a short distance (70 m) in her residency area, however, there were multiple angled and sharp turns to complete the path. The data collection time and GuideNav runtime was held on a different day and time, and various changes in the environment (e.g., parked car, a crowd of pedestrians passing by - jogging, riding bicycles, light changes) made VT&R more challenging due to appearance change. In total, six interventions were required for guiding the user over 4.9 km.

#### 7.4.1 Satisfying and Easy Outdoor Travel in Familiar Routes.

GuideNav enabled all BLV handlers to successfully complete their familiar community routes, ranging from 70 m to over 2 km round trip, with minimal interventions. Participants consistently expressed surprise that the robot was fully autonomous, even through routes with multiple sharp turns. As H01 remarked, *“Really it was a hundred percent itself? Even around plant planters, that was all on its own. Wow. It was a success.”* Similarly, H02 reflected after a repeat trial, *“So it really did know where it was going and we did do the route, wow.”* Walking with the robot was described as straightforward and low effort. H03 summarized, *“It seems like it’s got some potential for it, for sure. It wasn’t hard or anything. It was pretty easy.”* Both handlers and trainers highlighted the robot’s ability to negotiate uneven terrain. During observation of H03 walking with his guide dog, we noted surface tree roots. And later in the teach phase, we teleoperated the robot to safely maneuver over them, and in the repeat phase, the robot autonomously guided the user across the same spot. This led H03 to remark it was *“pretty seamless,”* and T01 to observe, *“Yeah, it actually avoided [them].”* Collectively, participants framed the experience as *“easy to walk with”* and emphasized its significance beyond laboratory trials. As H03 put it, *“I honestly think that either yours or this will be the future of blindness, the mobility of blindness.”* These reactions highlight not only the robot’s technical robustness but also its perceived potential as a practical mobility aid.

#### 7.4.2 Comparison with Guide Dogs:

*Trainers’ Perspectives.* Professional trainers evaluated our guide dog robot against standard guide dog assessment criteria, noting both promising capabilities and differences. The blindfolded trainer (T02) described the experience as sturdy and enjoyable, while the evaluator (T03) concluded, *“She looked comfortable. I thought it was very safe in its movements. I didn’t feel like she was going to run into anything.”* An experienced trainer (T01), who had previously seen the robot under manual control, remarked on its progress and usability: *“Compared to two years ago, the technology has come a long way as far as the actual usability of it in real life. I was impressed by its ability to navigate […] the concept of putting in a route and following it specifically was very impressive.”*

At the same time, T02 emphasized that the robot’s movement remained fundamentally different from that of a guide dog, describing it as more abrupt and less fluid: *“To put it very basically, the movement of the robot is just very robotic. It doesn’t have the organic flow that a dog does. Even when you’re approaching a curve with a dog, you can feel through the harness that the dog is slowing. With the robot, you get fewer of those body cues because the robot doesn’t have them.”*

Trainers emphasized the value of explicit indications for landmarks, turns, and stops to support user orientation. For example, after observing a blindfolded trainer (T02) guided by the robot, evaluator T03 suggested introducing short pauses before directional changes: *“If maybe when we got to that intersection of the sidewalk to pause for him to be stationary and then turn left, I think it would be a good point of orientation for [T02] to realize that we were making a directional change.”*

#### 7.4.3 Workload and Usability Evaluation.

The NASA–TLX instrument [28] results showed a clear divide between BLV handlers and professional trainers (Table 3). All three BLV handlers (H01–H03) reported very low workload (median TLX scores below 6 for mental, physical, and frustration), describing the robot’s guidance as requiring minimal cognitive or physical effort. In contrast, the blindfolded trainer (T02) rated workload much higher (48/100), reflecting both a stricter professional benchmark (guide dog expectations) and first-time use of the robot. One handler (H02) showed a large reduction in workload across sessions, with mental demand, effort, and frustration dropping substantially in her second trial, aligning with contextual factors – her first route was disrupted by neighborhood distractions, although it did not affect GuideNav’s performance.

System Usability Scale (SUS) scores also varied. Two handlers (H01, H03) rated the system extremely high (92.5 and 95.0), describing it as intuitive and effortless. H02 rated it much lower (32.5 → 40.0), reflecting initial difficulties and possibly reduced comfort due to her residual vision when told the robot would guide autonomously. The trainer (T02) rated the robot at 52.5, notably lower than her benchmark for a guide dog (80.0), underscoring a usability gap. Overall, BLV users experienced the system as easy and non-frustrating (low TLX, high SUS), while trainers applied stricter standards. These results suggest that GuideNav can achieve very high usability for some users, but evaluations must account for adaptation, personal context, and professional expectations.

## 8 Discussions and Conclusion

Our study shows that GuideNav can already deliver usable guidance on familiar community routes, yet several design refinements remain critical. Both handlers and trainers emphasized the need for smoother motion cues and explicit landmark indication (e.g., cues for curbs, crossings, or turns) to better align with orientation practices taught in O&M training. In our current implementation, GuideNav’s controller directly executes raw velocity commands, which can occasionally produce jerky motion. We plan to apply velocity smoothing via filtering to improve comfort and consistency. Future work will explore multimodal cues—audio, haptic feedback, or brief pauses—to signal turns and landmarks and better support user orientation, while also addressing bystander privacy concerns in vision-based navigation.

Beyond these improvements, trainers envisioned diverse applications: as a lower-maintenance alternative for those unable or unwilling to care for a guide dog; as a training tool for instructors to assess mobility skills, balance, and pace before matching a person with a guide dog; as a temporary substitute when users are between guide dogs; and as a flexible solution for pre-planned routes in settings such as hospitals, conferences, or community centers. Because the system requires only a single camera, it could be embodied in multiple robot forms and deployed at scale, broadening access for both cane users and guide dog users. Together, these findings highlight not only the feasibility of vision-only teach-and-repeat navigation, but also its potential to fill important gaps in assistive mobility, opening new directions for human-centered guide robot design.

## Supplementary Material

video attachment

## Figures and Tables

**Table 1:** User Study Participant Demographics

ID	Age	Gender	Vision Level	Experience*	Role

H01	63	F	Totally blind	35	User
H02	66	F	Legally blind	9	User
H03	67	M	Totally blind	15	User

T01	37	F		10	Evaluator
T02	35	F		4	User
T03	28	F		7	Evaluator

* Indicates the number of total years the participant trained guide dogs.

**Table 2:** Robot Solo and Handler-Guide Dog Robot Team Navigation Results

Trajectory ID	Environment		Performance		
	# TSR	Distance (m)	# Collisions	# Interventions	Time

Robot Solo (FidelcoFront → Storage)	5/6	130 m	0	2	4 min 8 s
Robot Solo (FidelcoFront → Parking)	6/7	160 m	0	1	4 min 2 s
Robot Solo (LongWalkLoop)	17/17	1670 m	0	1^†‡^	44 min 58 s
Robot Solo (ResidentialLoop)	13/14	430 m	1	3	31 min 12 s
Robot Solo (ClassroomLoop–Indoor)	4/6	30 m	0	3*	2 min 42 s

**Total**	45/50 (90%)	2420 m	1	10	87 min 2 s

H01+Robot (Lot → Library)	4/4	390 m	0	0	12 min 14 s
H01+Robot (Library → Lot)	3/3	390 m	0	0^†^	12 min 54 s
H02+Robot (Home → BusStop)	7/7	70 m	0	0	3 min 10 s
H02+Robot (Dining → FineArts)	3/3	670 m	0	0	13 min 43 s
H02+Robot (FineArts → Dining)	2/2	670 m	0	1^†^	18 min 6 s
H03+Robot (Home → Church)	8/8	1010m	0	1^‡^	21 min5 s
H03+Robot (Church → Home)	8/8	1010m	0	3^‡^	24 min 3 s
T02+Robot (Guide dog evaluation loop)	11/11	730m	1	1^‡^	18 min 57 s

**Total**	46/46 (100%)	4940 m	1	6	124 min 12 s

† Technical malfunction (camera disconnection/overheating) - excluded from intervention count

‡ Single guidance intervention required for unexpected obstacle avoidance;

* Single physical intervention required to remove entangled cables from robot leg; TSR stands for Turning Success Rate

**Table 3:** Perceived workload (raw 0–20 NASA-TLX scores)

Component	H01	H02	H03	T02	Median

Mental demand	4	5 → 4	8	10	6.25
Physical demand	1	1 → 2	5	10	3.25
Temporal demand	2	0 → 0	5	5	3.50
Performance	1	0 → 5	5	4	3.25
Effort	2	5 → 2	7	16	5.25
Frustration	1	10 → 4	1	3	2.00

**Total**	11	21 → 17	31	48	25.00

## CCS Concepts

- **Human-centered computing** → **Field studies**; **Accessibility technologies**; **• Computer systems organization → Robotic autonomy**; **• Computing methodologies** → *Vision for robotics*
